# Health consequences of drought in the WHO Eastern Mediterranean Region: hotspot areas and needed actions

**DOI:** 10.1186/s12940-020-00665-z

**Published:** 2020-11-12

**Authors:** Saverio Bellizzi, Chris Lane, Mohamed Elhakim, Pierre Nabeth

**Affiliations:** Emergency Department, World Health Organization, Eastern Mediterranean Regional Office, Cairo, Egypt

**Keywords:** Drought, Eastern Mediterranean region, Preparedness, World Health Organization

## Abstract

**Background:**

Over the past four decades, drought episodes in the Eastern Mediterranean Region (EMR) of the of the World Health Organization (WHO) have gradually become more widespread, prolonged and frequent. We aimed to map hotspot countries and identified key strategic actions for health consequences.

**Methods:**

We reviewed scientific literature and WHO EMR documentation on trends and patterns of the drought health consequences from 1990 through 2019. Extensive communication was also carried out with EMR WHO country offices to retrieve information on ongoing initiatives to face health consequences due to drought. An index score was developed to categorize countries according vulnerability factors towards drought.

**Results:**

A series of complex health consequences are due to drought in EMR, including malnutrition, vector-borne diseases, and water-borne diseases. The index score indicated how Afghanistan, Yemen and Somalia are “hotspots” due to poor population health status and access to basic sanitation as well as other elements such as food insecurity, displacement and conflicts/political instability. WHO country offices effort is towards enhancing access to water and sanitation and essential healthcare services including immunization and psychological support, strengthening disease surveillance and response, and risk communication.

**Conclusions:**

Drought-related health effects in the WHO EMR represent a public health emergency. Strengthening mitigation activities and additional tailored efforts are urgently needed to overcome context-specific gaps and weaknesses, with specific focus on financing, accountability and enhanced data availability.

## Introduction

In the World Health Organization (WHO) Eastern Mediterranean Region (EMR), with around three quarters of its surface consisting of desert, drought, defined as a prolonged dry period in natural climate cycle, is a common phenomenon [1].

Over the past four decades, drought episodes in the EMR have gradually become more widespread, prolonged and frequent [2]. The drought that lingered between 1998 and 2012 was likely the worst of the past nine centuries for countries like Jordan, Lebanon, Palestine and Syria [2]. In particular, the 4-year drought that started in 2006 in Syria has had major consequences with mass migration from the countryside to the cities [2].

The Horn of Africa has been affected by prolonged drought in 2016 and 2017 and has been identified as an important vulnerability area along with the south-western Arabian Peninsula (Yemen) [3]. Similarly, large scale drought conditions erupted in selected and contiguous provinces of Afghanistan and Pakistan during the last trimester of 2018 [3].

**Fig. 1:**
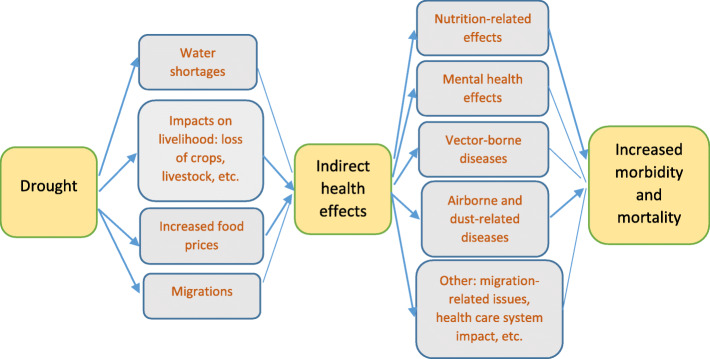
Impact of drought on health

Being a slow-onset, long duration, spatially diffuse emergency, rather than a sudden, high-impact event (such as flash flood), drought differs from other natural hazards and has many multiple “downstream” effects that might result in increased morbidity and mortality (Fig. 1) [4].

The nutrition-related morbidity and mortality are the best recognized health impact of drought [5]. Drought affects ecosystem, thereby reducing food supplies like crops and livestock, and its related food shortage is only one aspect of the much more important concept of food security (defined as ... physical and economic access to sufficient, safe and nutritious food to meet dietary needs.) [6].

Decreased water availability is a defining feature of most drought. As water levels are typically low, this may lead to both reduced dilution capacity and to contamination of the few remaining sources. The likelihood for an outbreak of infectious disease (e.g. Cholera) to occur increases when more users are there for a water source [4]. Similarly, skin infections are common because of lack of water for washing and include scabies and leishmaniasis.

On the other hand, as soils become increasingly dry during a drought, dust circulated in the air is more likely. Dust, in its turn, can be harmful via two mechanisms: pathogen carriage and direct trauma from inhaled particulates [5].

It is also important to note that density of mosquitos, among the most important arthropod vectors involved in the transmission of various vector-borne pathogens, has been found to dramatically increase following natural drought events due to the loss of competitors and predators [7].

Also, the proliferation of water storage tanks as an adaptation strategy during drought conditions, may create a potential for transmission of diseases like dengue [8], chikungunya [9] and Rift Valley fever [10].

Research suggests that drought contributes to the business-related pressures that farmers must face, with severe drought resulting in financial impacts and consequent emotional stress [4].

Finally, effects of flooding in the area subjected to drought are particularly devastating because of rainfall not being absorbed into the soil to nurture crops [4].

The factors linking drought with effects on health occur within the context of existing infrastructure (i.e., health, sanitation and other resources) and baseline public health (i.e., the capacity of populations to be resilient in the face of adverse conditions) [4].

In consideration of all the ongoing as well as future potential adverse health outcomes of drought, a public health situational analysis was conducted with the overall objective of guiding the World Health Organization towards preparedness actions in the Eastern Mediterranean Region. More specifically, this analysis was guided by the need of: I) Assessing the health consequences of drought and their vulnerability characteristics (i.e. food security, water, sanitation and hygiene) at EMR level; II) Mapping hotspot countries for health consequences according to these vulnerability characteristics; and III) Identifying the key strategic actions for WHO actions against health consequences of drought.

## Methods

### Health consequences of drought and vulnerabilities in WHO EMR

A review of scientific literature and WHO EMR documentation indicating updated trends and patterns of the drought health consequences from 1990 through 2019 was carried out. To do so, we relied on publically available articles in PubMed, on data displayed on the WHO Regional Health Observatory, on EMR specific programmes documents, and on WHO Country Office reports.

In the context of drought, vulnerability is a function of three major drivers, including exposure,

sensitivity and adaptive capacity. Specifically, vulnerability has different dimensions and is affected by economic, socio-culture, psychological, technical and infrastructural factors [11, 12].

In this regard, the World Health Organization lists specific vulnerable factors [1], which we took into consideration when reviewing all updated specific documentation to explore the regional health impact of drought.

The criteria were: health status of the population before the disaster; infrastructure like water supply and sanitation systems; food insecurity; absence of warning systems; population displacement; and other concurrent situations like economic crisis, political instability and armed conflict.

The specialized WHO Centre for Environmental Health Action (CEHA) was contacted to provide relevant documentation on access to water and sanitation in the EMR countries.

### Drought health consequences hotspot map

In order to map the burden of health consequences due to drought in the EMR, an index score, which that takes into account all the above-mentioned vulnerability factors, was developed, and all EMR countries were assessed against them after categorization. Targets defined under the United Nations Sustainable Development Goals and other globally used variables were considered and categorization, if not binary, followed standard threshold distribution of values as follows:

Under-5 mortality [13] (U5M) was used as proxy for the health status of the population and dichotomized, in line with the Sustainable Development Goal (SDG) 3.2, in “0” if U5M rate was less than 25 deaths per 1000 livebirths and “1” if otherwise.

Even if SDG 6.2 target is 100% of people have access to adequate and equitable sanitation and hygiene, we have considered 3 levels of access: countries were coded as “0” if more than 90% of the population had access to basic sanitation, “1” if this proportion was between 50 and 90%, and “2” if otherwise [14].

To estimate the food insecurity [15] status, we used the estimated population in need of emergency food assistance in 2019, and considered the four categories proposed by the Famine Early Warning System (FEWS) maps [15]. Countries with population in need accounting for less than 1 million, between 1 million and 5 million, between 5 million and 15 million, and for more than 15 million, were coded as 1, 2, 3, and 4 respectively.

Countries were assigned a code equal to “0” if no Early Warning System for drought was in place and “1” if otherwise.

Last vulnerability factor considered was the internal displacements, using UNHCR figures on Internally Displaced Persons (IDPs) [16]. Countries were coded as “1” if they had less than 1 million IDPs, “2” if IDPs were between 1 and 5 million, and “3” if they were more than 5 million IDPs).

For each country, an index score (from “2” up to “11”) was calculated after summing all the vulnerability factors’ scores. Hotspots were identified as countries with the highest scores for health consequences due to drought.

In line with the described index score, an EMR map was produced with colours reflecting the status of health consequences vulnerability due to drought.

### WHO actions

Extensive communication with all EMR WHO country offices was conducted to retrieve information on current and ongoing initiatives to face health consequences due to drought at country level. Particular emphasis was put to identify gaps and weaknesses as well as needed interventions.

## Results

**Fig. 2:**
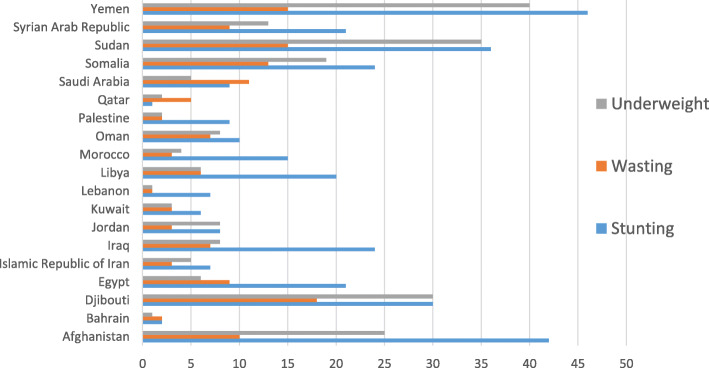
Distribution of prevalence of stunting, wasting and undernutrition by country in the EMR, in 2019

Malnutrition is a complex challenge for the Eastern Mediterranean Region, with many countries having multiple forms of malnutrition among their populations at the same time. Populations are mainly affected by undernutrition (wasting, stunting, underweight) [17]. The overall estimates for stunting, wasting and underweight are 28, 8.7 and 18% respectively (Fig. 2) [17]. The prevalence of anaemia (haemoglobin < 11 g/dl) that is the result of iron deficiency ranges from 7.4 to 88% in children aged < 5 years and from 16 to 81% in pregnant women [17].

Decreased water availability and extensive population movements across and within the countries, especially in areas with porous borders like the Horn of Africa and from the Sahel to the Northern African countries, make difficult the identification of trajectories of transmission of water-borne diseases like cholera which have affected several EMR countries over the last years. The cumulative number of suspected cholera cases reported in Yemen since 2017 until the end of 2019 was 2,188,503 3750 associated deaths, resulting in a 0.17% case fatality rate [18]. Acute Watery Diarrhea (AWD) had affected Sudan since 2016 infecting over 36,000 people and killing over 800 [19]. Somalia is highly endemic for cholera and regular large outbreaks both after flooding and during droughts are observed [20]. Cholera outbreaks also occur in Iraq every 3 to 5 years, with last considerable outbreak occurring in 2015, and have a distinct seasonality that typically start in September and continue through December [21].

Up to 60% of the worldwide burden of cutaneous leishmaniasis is in the EMR [22]. Massive destruction of urban settlements during the crises, poor waste management, limited access to safe and clean water, presence of domestic animal that act as the reservoir for the sandflies and lack of effective vector control program put large number of people at risk of Leishmaniasis in the region [22].

The geographic diversity in the EMR determines malaria variability in terms of endemicity, intensity of transmission and type of malaria. Malaria-endemic countries of the region are situated in the three eco-epidemiological zones of malaria: Afrotropical, Oriental and Palearctic [23, 24].

In Saudi Arabia, Yemen and the sub-Saharan countries of the region (Djibouti, Somalia, Sudan), *P. falciparum* is predominant. In the other endemic countries, mainly Afghanistan, Islamic Republic of Iran and Pakistan, both *P. falciparum* and *P. vivax* are transmitted [23]. More than 20,000 have been recently reported in Djibouti with the introduction of a new vector.

The under-five mortality ranges from less than 10 deaths per 1000 live births in in the Gulf countries and Lebanon, up to more than 50 deaths per 1000 live births in Afghanistan, Djibouti, Pakistan, Somalia, Sudan and Yemen. The same pattern is present for the neonatal mortality, with Afghanistan, Pakistan and Somalia reaching highest incidence (> 35 deaths per 1000 live births) [13].

**Fig. 3:**
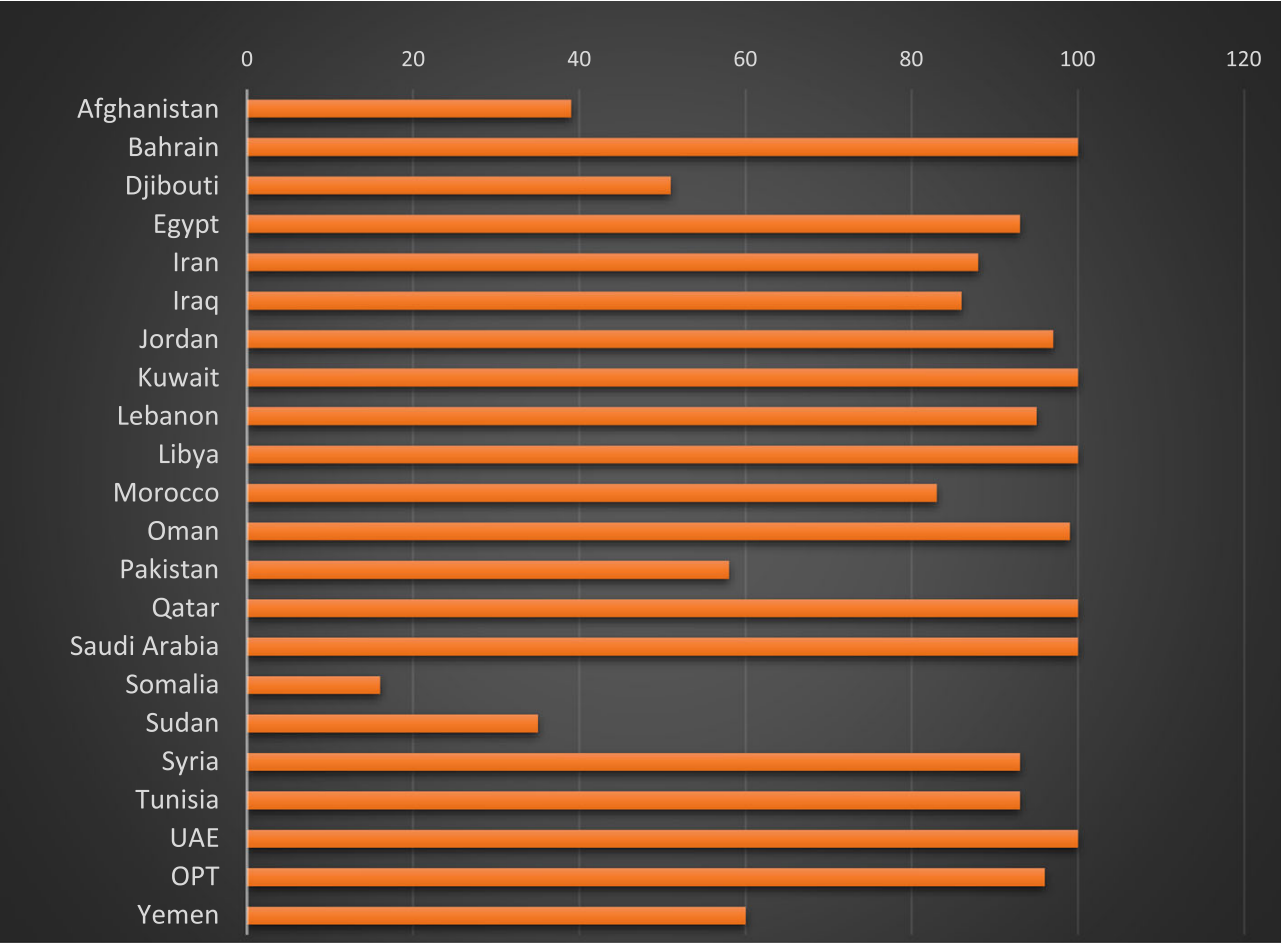
Country percentage of basic drinking-water services in EMR as of 2019

Eighty seven percent (564.08 million) of the Region’s total population has access to at least basic drinking-water services. Thirteen percent (84.4 million people) remain without even basic water services, of which 64.6 million live in Afghanistan, Pakistan, Sudan, Somalia and Yemen and 15 million live in Iraq, Islamic Republic of Iran and Morocco (Fig. 3) [25].

Estimates for safely managed drinking-water are available for only 9 out of 22 countries of the EMR (Bahrain, the Islamic Republic of Iran, Jordan, Kuwait, Lebanon, Morocco, Oman, Pakistan and Tunisia). Bahrain, Islamic Republic of Iran, Kuwait, Jordan and Tunisia have the highest percentage of the population (over 89%) with access to safely managed water services [25].

**Fig. 4:**
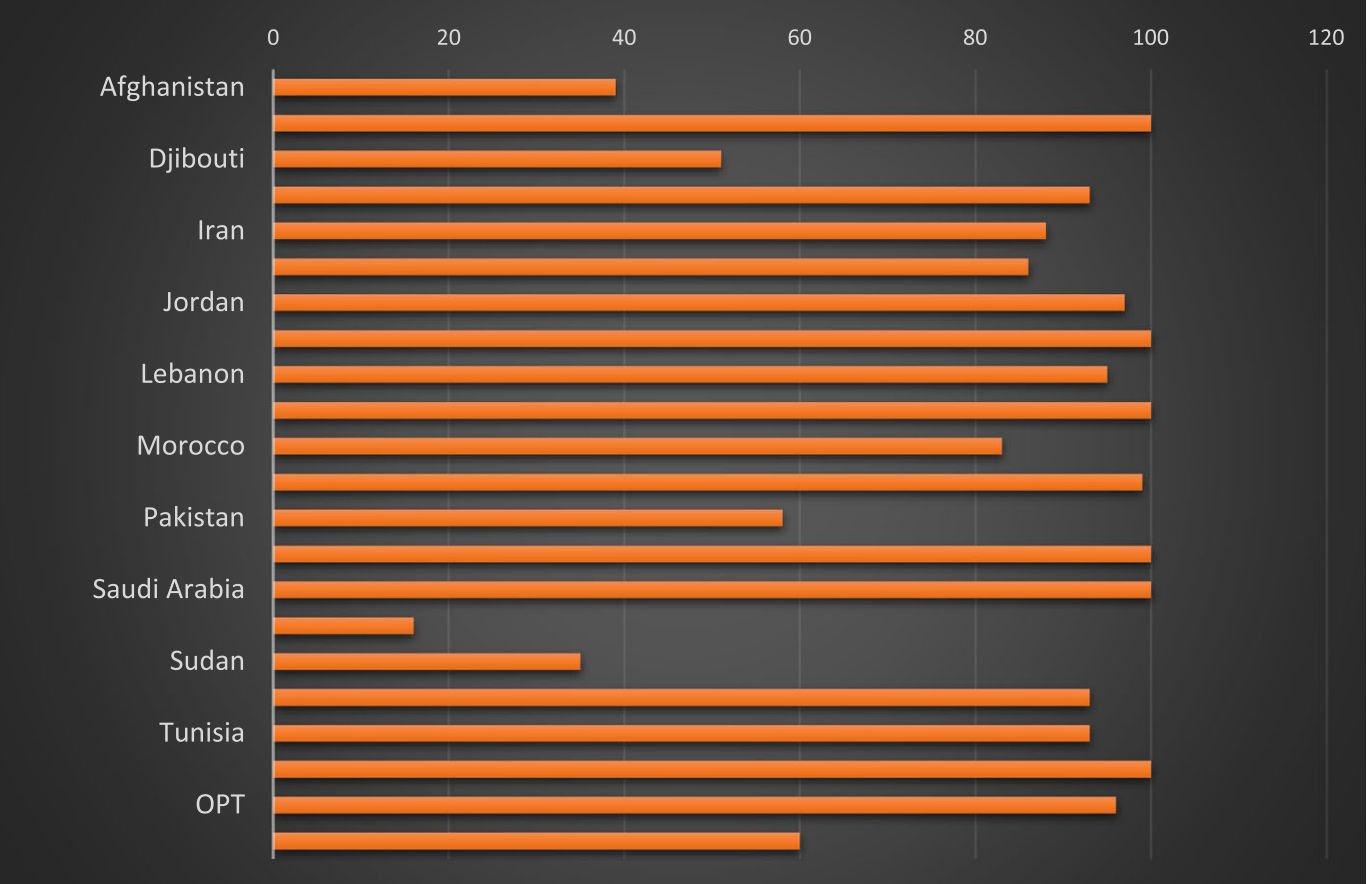
Country percentage of basic sanitation services in EMR as of 2019

Twenty seven percent (175 million people) remain without basic sanitation services, of which 154 million live in Afghanistan, Islamic Republic of Iran, Pakistan, Somalia, Sudan and Yemen and 17 million live in Egypt, Iraq and Morocco (Fig. 4) [25]. Around 51.7 million people in the region still defecate in the open, mainly in rural areas, of whom 46.5 million live in Afghanistan, Pakistan, Somalia, Sudan and Yemen. Access to water and soap for handwashing varies greatly, ranging from 10% in Somalia to around 90% in Tunisia, Egypt and Iraq [24].

The humanitarian food assistance continues in Yemen through early 2019, especially for the significant declines in commercial imports and conflict that cuts populations off from trade and humanitarian food assistance for an extended period, potentially leading to Famine [26].

For Afghanistan, the Famine Early Warning Systems Network (FEWS NET) estimates that the overall population in need of emergency food assistance in 2019 is between 5 and 10 million [15].

Pakistan has made gains becoming a food surplus country, and a major producer of wheat. However, around 2 million persons are estimated to be in need of emergency food assistance [15], primarily due to limited economic access by the poorest and the most vulnerable.

The collapse of the economy, soaring food prices, loss and disruption of livelihoods as well as the decline in food production have contributed to widespread food insecurity across Syria. More than 5 million Syrians are now estimated to be in need of emergency food assistance [13]. Similar dynamics in the neighboring Iraq have led to around 2 million people in need for the year 2019 [15].

Well above average staple food prices are expected to drive high 2019 assistance needs in Sudan, and more than 5 million population is now estimated to be in need of emergency food assistance [15].

Countries in the EMR are at different stages as to the presence of functioning drought early warning systems. While a dedicated regional network is well set-up for most of the Arabian Peninsula countries and for northern African countries like Morocco and Tunisia [27], other countries like Iran rely on government centers. Afghanistan and Somalia rely on specific donors funded projects [28] while all the other countries do not have specific monitoring initiatives.

As host to some of the world’s biggest emergencies and protracted crises, the EMR carries the largest burden of displaced population globally [29]. Out of 58 million displace persons worldwide, almost 30 million (52%) come from the Region. As far as internally displaced persons (IDPs) are concerned, Syria reaches the peak of around 6 million IDPs, followed by Afghanistan (around 2 million), Iraq, Somalia, Sudan and Yemen; Libya and Pakistan feature (around 200,000 IDPs each) [16].

The conflict in Yemen is marked by severe blockades to humanitarian access including aerial and naval blockade of humanitarian goods. Import blockage to food, fuel and medicine have directly impacted on nutritional status, water, sanitation and hygiene (WASH), and health care of the population [30].

Several decades of conflict and insecurity have led to extensive degradation of infrastructure and public services across all sectors in Somalia [31].

The new ongoing conflict in Libya has caused several casualties including health care workers [32]. Similarly, occupied Palestinian territory (oPt), Syria and Afghanistan struggle to provide health care services in insecure and under-resourced settings [33].

**Fig. 5:**
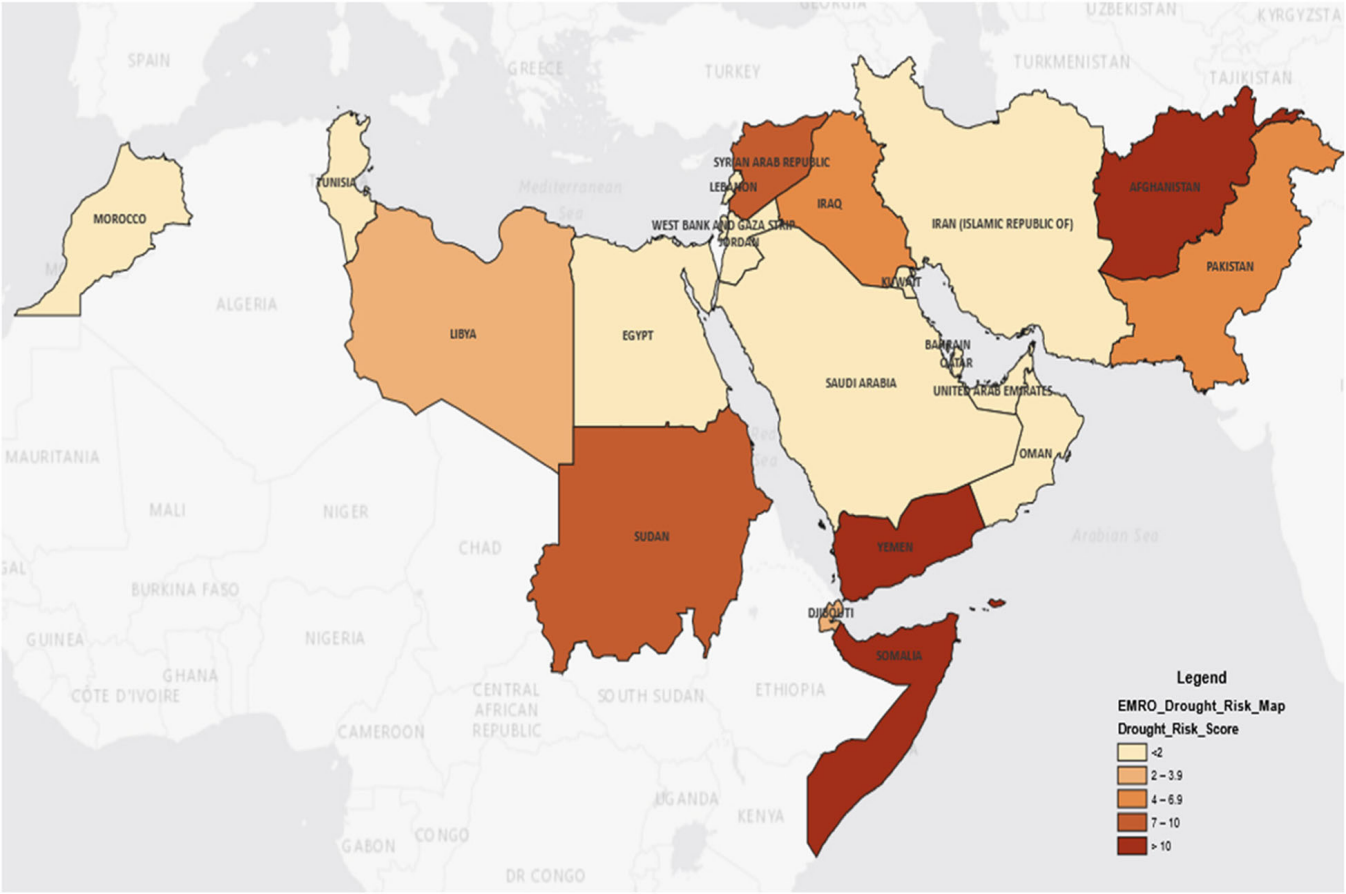
Hotspot map for health consequences due to drought in the WHO EMR

The health consequences vulnerability index score yielded well defined-areas at risk for drought. The below map indicates how Afghanistan, Yemen and Somalia are “hotspots” due to poor population health status and access to basic sanitation; other elements like high food insecurity, displacement and the conflicts/political instability render these contexts further vulnerable (Fig. 5).

## Discussion

The WHO Eastern Mediterranean Region remains a highly vulnerable area of the world in terms of health consequences due to drought. This public health situational analysis highlights how several health consequences are either endemic (malaria) or being present for decades (undernutrition) and worsening because of impeding additional threats like drought. Vulnerabilities play a fundamental role in the mitigation process against the potential aftermath of drought and vary greatly across countries of the EMR. Therefore, it is not surprise that countries like Afghanistan, Somalia and Yemen feature as hotspots for morbidity and mortality due to health consequences of drought. These findings are even more alerting when drought forecast assessments are taken into considerations. The United Nations Economic and Social Commission for Western Asia (UNESCWA) integrated vulnerability assessment [34] clearly shows how vulnerability hotspots for drought generally recur in the Sahel extending northward into the Sahara Desert, the south-western Arabian Peninsula, and the Horn of Africa, particularly for the high exposure to comparatively larger increase in temperature and declining precipitation.

It is important to emphasize how such territories are characterized by the important representation of vulnerable groups like children, particularly at risk because of their cognitive and physical development, socioeconomic status and access to basic health resources [35, 36]. Also, we cannot underestimate the effect of unavailability of water on the health of women and adolescent girls due to compromised maternity health services as well as compromised personal hygiene practices. On the other hand, the effects of drought have potentially greater impact on individual with underlying chronic medical conditions such as respiratory diseases as well as on persons with disabilities, who may not be able to access emergency response services due to the difficulties in mobility, hearing, seeing and understanding.

As previously mentioned, drought tend to force people to migrate with the hope of better conditions [37]. However, these migration dynamics can cause more health problems because of the tendency for transmittable diseases to spread [38].

Evidence suggests that the greater the impact of drought, the more likely the drought will increase vulnerability to the next extreme event, particularly another drought or flood in low-resourced settings if there is no adequate time for the individual and community to recover [39]. One study from Brazil found that semi-arid regions across 20 years made slower gains in population health when compared to the other regions of the country; namely, infant mortality rate, poverty, illiteracy and life expectancy regularly remained worse off [40].

To make things worse, drought usually co-occurs with heat waves and wildfires at some places and that makes the health outcomes more complicated and the response to these disasters more challenging. Compounding or cascading dry hazards are expected to have more negative impacts than each of the hazards alone. For instance, the drought that occurred in 2003 was not the most severe in Europe. However, in combination with extended heatwaves and fires, it is considered as the most fatal and costly with more than 70,000 people passed away and an economic damage exceeding 8.7 billion euros [41].

Another crucial consideration for the Eastern Mediterranean Region is the interaction between food security and climate events in natural and human-induced disasters or political instability [41]. Protracted crises are the new norm, especially in EMR, with significant implications for vulnerability to extreme weather and climate events [42]. In 2012, approximately 366 million people lived in protracted crisis situations, of whom approximately 129 million were undernourished (around 19% of the global total of food-insecure people) [42].

As reported by Yusa et al., all Global Climate Models (GCMs) project future increases of summer continental interior drying and associated risk of droughts, with this particularly applicable to areas like the Mediterranean region [43]. It is also important to emphasize the synergic role of climate change towards the health risks posed by droughts: while rising sea levels can threaten freshwater supplies for people living in low-lying areas, more severe storms can cause city sewage systems to overflow leading to increase in communicable diseases. Similarly, while more heat can mean longer allergy seasons, more rain increases mold, fungi, and indoor air pollutants, thus aggravating respiratory disease.

The World Health Organization is currently supporting several countries by strengthening the coordination of humanitarian response at central and provincial level. Humanitarian response involves also coordination with partners focusing on the provision of adequate quantities of clean water and improvements in sanitation.

Despite public health emergency officers have been recruited in different settings to coordinate implementation of response activities, additional support is required for the monitoring and evaluation of response activities; this is for instance the case of Somalia where the situation is complicated by political and administrative differences across states.

In Afghanistan, several mobile health teams delivering integrated health, nutrition and psychosocial services were deployed for drought affected IDPs. However, the number of mobile teams, including those to refer critical patients to nearest health facilities, has recently declined because of shortfall of fund. Funds is also requested in Somalia for training and deployment of Integrated Emergency Response Teams in hard-to-reach areas. While specific preparedness plan for Cholera exist in countries like Somalia, Sudan and Yemen, funds are again critical for accelerated vaccination activities in drought-prone areas. On the other hand, well-tailored activities for other epidemic diseases like measles are partially existing; this is the case of Somalia where there is need of training of health care providers on measles case management. In parallel, rapid assessment of the routine Expanded Programme on Immunization (EPI), considering the lower EPI coverage and the tendency for the measles outbreaks in drought affected areas, is routinely carried out. Training of health workers in the management of essential medicines for epidemic prone diseases is another common gap across countries. Mental health support is widely acknowledged to be largely neglected in the EMR area and preparedness actions include the training of staff and procurement of mental health drugs. Warehousing of medical supplies is another generalized gap, like in the case of Iraq, and deserves context-specific strategies.

While early warning surveillance systems are in place in various areas, expansion of current platforms to adequately cover drought-prone areas with training of health workers in early surveillance use as well as on investigation of alerts (rapid response teams) is needed. Quality of data and irregular reporting remains an issue like in the remote areas of Iraq among others.

Training is also required in water quality assessment for health workers in addition to the procurement of water testing kits.

In Afghanistan, health education is part of the Ministry of Health responsibilities, particularly for pregnant women in order to be encouraged to give birth at the health facility. However, more needs to be done to communicate specific risks exposed to the IDPs. Communication messages of specific risks for epidemic prone diseases must be developed in local languages with alignment to country communication strategy.

Indeed, a series of challenges must be seriously taken into consideration when planning support at country level. Insecurity is a major impediment for the humanitarian actors to deliver assistance especially in countries such as Somalia, Afghanistan and Yemen. All parties in the ongoing conflict show lack/insufficient adherence to the international humanitarian law, and insufficient willingness to fulfil their responsibilities in respect to protection of civilians and health workers. Health workers and programmes have been targeted by different parties to the conflict or becoming “collateral damage”. There is very difficult to find qualified staff willing to work in areas affected by conflicts. The insecurity affects not only the implementation but also the capacity to conduct rapid assessments, verification of data and monitoring.

Lack/ insufficient reliable data and difficulties in conducting assessment are also of concern. There is a need to increase the feasibility of conducting assessments, mapping of resources and capacities across the country. Collaborative agreements with partners that have access in difficult areas could be a solution.

Difficulty in monitoring that the assistance supported the intended beneficiaries is another issue faced during disasters. Pre-disaster local agreements with partners and networks well established and accepted by communities such as polio focal points and Red Crescent Society volunteers could help over passing this problem.

Weak government provincial/ district structures as a result of insufficient resources, staffing and technical capacities, also limits the availability and quality of assistance delivered. Other issues are linked to misuse of available resources due to lack of accountability, insufficient coordination and duplication.

Drought is well represented among the priorities embedded in the Sustainable Development Goals, such as SDG 13 (Climate actions) and SDG 15 (Life on land). To further support countries and populations to reach SDG, actions like raising awareness on water right and water saving, investing in sustainability science research for drought and strengthening resilience efforts via international cooperation are key to enhancing drought resilience and preparedness [44].

## Conclusions

Drought-related health effects in the WHO Eastern Mediterranean Region represent a public health emergency due to the baseline susceptibility in countries like Yemen, Somalia and Afghanistan. Several other countries result at high risk of health consequences due to displacement, poor health and sanitation, which add to the complex protracted crises. Strengthening mitigation activities is a priority for the WHO Regional Office of the Eastern Mediterranean Region and multifaceted initiatives are ongoing. However, additional tailored efforts are needed to overcome context-specific gaps and weaknesses. This would include increased financing and accountability, enhanced data reliability and adequate staffing and training of health operators in the field.
